# Suppressing an Anti-Inflammatory Cytokine Reveals a Strong Age-Dependent Survival Cost in Mice

**DOI:** 10.1371/journal.pone.0012940

**Published:** 2010-09-23

**Authors:** Virginia Belloni, Bruno Faivre, Romain Guerreiro, Emilie Arnoux, Jérôme Bellenger, Gabriele Sorci

**Affiliations:** 1 BioGéoSciences, Unité Mixte de Recherche 5561, Centre National de la Recherche Scientifique, Université de Bourgogne, Dijon, France; 2 Physiopathologie des dyslipidémies, Unité Mixte de Recherche U866, Institut National de la Santé et de la Recherche Médicale, Université de Bourgogne, Dijon, France; New York University, United States of America

## Abstract

**Background:**

The central paradigm of ecological immunology postulates that selection acts on immunity as to minimize its cost/benefit ratio. Costs of immunity may arise because the energetic requirements of the immune response divert resources that are no longer available for other vital functions. In addition to these resource-based costs, mis-directed or over-reacting immune responses can be particularly harmful for the host. In spite of the potential importance of immunopathology, most studies dealing with the evolution of the immune response have neglected such non resource-based costs. To keep the immune response under control, hosts have evolved regulatory pathways that should be considered when studying the target of the selection pressures acting on immunity. Indeed, variation in regulation may strongly modulate the negative outcome of immune activation, with potentially important fitness consequences.

**Methodology/Principal Findings:**

Here, we experimentally assessed the survival costs of reduced immune regulation by inhibiting an anti-inflammatory cytokine (IL-10) with anti-IL-10 receptor antibodies (anti-IL-10R) in mice that were either exposed to a mild inflammation or kept as control. The experiment was performed on young (3 months) and old (15 months) individuals, as to further assess the age-dependent cost of suppressing immune regulation. IL-10 inhibition induced high mortality in old mice exposed to the mild inflammatory insult, whereas no mortality was observed in young mice. However, young mice experienced a transitory lost in body mass when injected with the anti-IL-10R antibodies, showing that the treatment was to a lesser extent also costly for young individuals.

**Conclusions:**

These results suggest a major role of immune regulation that deserves attention when investigating the evolution of immunity, and indicate that the capacity to down-regulate the inflammatory response is crucial for late survival and longevity.

## Introduction

Costs and benefits of the immune response have attracted considerable attention in the last years among evolutionary biologists [1–3 for reviews]. Ecological immunology considers the immune response as a trait whose expression should be optimized by natural selection as to maximize fitness. Therefore, the central paradigm of ecological immunology follows an economical perspective and postulates that immune defences are beneficial, but also costly, and that natural selection should minimize the ratio between costs and benefits [2], [3]. Costs occur when investment into immune defences is afforded at the expense of other fitness-related functions, generating trade-offs between competing traits [1], [2]. Most ecological immunology studies have focused on such resource-based costs of immunity [4], [5], and with a few exceptions [6]–[9], have neglected resource independent costs. This is surprising, given the potentially devastating costs of autoimmunity. Indeed, the immune system can be depicted as a two-edged sword where one edge protects against infectious diseases and the other edge exposes to the risk of misdirected or over-reacting immune responses [10]. This phenomenon is generally called immunopathology and illustrates the process of immune system attacking self-structures of the host. Classical examples of immunopathology are those due to inflammation, a crucial component of the immune system. Several diseases arise as collateral undesirable short-term or delayed outcomes of acute and chronic inflammation, especially in advanced age [11]–[14].

Since an over-reacting immune response, or a response failing to make a distinction between self and non-self, potentially generates substantial fitness costs, organisms have evolved regulatory mechanisms based on cells and effectors whose function is to control the immune system and dampen the negative consequences of its activation. Therefore, in addition to the selection pressures acting on the allocation pattern of energy/resources to immune organs and cells, it seems plausible to expect strong selection on the regulatory mechanisms that prevent the system to get out of control. However, in spite of its importance, immune regulation has been neglected in studies devoted to the evolutionary forces shaping immune functioning. Here, we wished to assess the cost of suppressing the regulatory mechanisms underlying the control of the inflammatory response.

Inflammation is a non-specific process characterized by the delivery of effectors from the blood into infected tissues, and whose function is to fend off invading pathogens. At the systemic level, the inflammatory response induces fever, an increased number of leucocytes recruited for defence, and elevated levels of pro-inflammatory cytokines driving the microbicidal activity of phagocytic cells [10]. This category of immune cells produces cytotoxic compounds such as enzymes, lytic peptides or reactive oxygen and nitrogen species that kill pathogens [15], [16]. Inflammation is characterized by its rapidity (it occurs within minutes and hours after the encounter with a pathogen) and endows organisms with potent weapons to cope with infection. The most compelling support to this view is the high sensitivity to infection, and the drastically shortened survival prospect, of organisms with a defective inflammatory response [15]. However, because the compounds delivered during the inflammatory response do not discriminate between host and pathogen structures, they can potentially damage host's cells and tissues. Therefore, an over-expressed or misdirected inflammation may also inflict collateral damage to the host, and there is now extensive evidence showing that the cost of infection may be due to inflammatory-borne damage more than to a direct effect of the pathogen [17], [18].

To protect their structures from inflammatory injury, hosts have evolved regulatory mechanisms based on specific cytokines (e.g., interleukin-10, IL-10) controlling the resolution of inflammation. There exists a polymorphism in genes that code for such regulatory mechanisms [19], [20] and environmental effects (such as pathogen exposure, individual age) can, as well, shape the pattern of immune regulation.

In this study, we inhibited an anti-inflammatory cytokine in order to assess the cost of reduced immune regulation in mice. We used a full factorial design with three factors: suppression of an anti-inflammatory cytokine [injection of anti-IL-10 receptor antibodies (anti-IL-10R) *vs*. rat IgG1 antibodies (IgG)], stimulation of the inflammatory response [injection of an *E. coli* lipopolysaccharide solution (LPS) *vs*. phosphate buffered solution (PBS) injection], age (3 *vs*. 15 month old).

## Materials and Methods

### Ethics Statement

The experiment has been conducted in compliance and has received the agreement of the Animal Care and Ethical Committee of the Université de Bourgogne, Dijon (protocol # 6904).

### Animals and reagents

Sixty young (3 months old) and sixty old (15 months old) virgin male C57BL/6 mice were purchased from Janvier (Laval, France). Upon arrival at the laboratory, animals were individually housed in Plexiglas cages and kept in an air-conditioned room (temperature 21±1°C, relative humidity 60±10%) with a 12-hours light/dark cycle. Pellet food and tap water were provided *ad libitum*.

### Treatment procedure

Young and old mice were randomly assigned to one of four groups (15 individuals per treatment). At day 0, the group “anti-IL-10R” received an intraperitoneal injection of 20 µg of monoclonal anti-IL-10 receptor antibodies (1B1.3a; BD PharMingen), whereas the group “IgG” received an intraperitoneal injection of 20 µg of rat IgG1 antibodies (Sigma) for control. At day 1, half of the males in each group were intraperitoneally injected with a solution of lipopolysaccharides (LPS) from *Escherichia coli* (serotype 055:B5, Sigma) at a dose of 0.05 mg/kg, whereas the other half were injected with a phosphate buffered solution (PBS) for control. This LPS dose is more than 500-fold and 30-fold lower than the 50% lethal dose of LPS in 2-month and 24 month old mice, respectively [21]. No injection was applied at day 2. This injection scheme was repeated three times, each three days, and the entire experiment covered a 9 day period.

Blood samples were obtained by retroorbital puncture immediately before the first injection (day 0) and 24 hours after the last injection (day 8). Body mass (±0.1 g) was measured at day 0, 2 and 8. Mortality rate was recorded every day for the duration of the experiment.

### Serum amyloid A quantification

Serum amyloid A (SAA), a protein of the acute phase response, was quantified using an immunoassay kit (Mouse SAA, KMA0011, Invitrogen). Coloration of the antibody complexes was measured with a spectrophotometer (spectramax plus 384, molecular device) at 450 nm.

### Statistical Analyses

Differences in survival rate were analyzed with a Log-rank test. We also used a log-linear model where in addition to the treatment we included changes in body mass as a potential predictor of survival rate. Two 15-month old male died during the first injection and were therefore removed from the survival analysis.

Generalized linear mixed models were used to assess the effect of the treatments and age on changes in body mass and SAA, with individual identity declared as a random variable. We started with the full model and then we dropped all non significant three- and two-way interactions. Degrees of freedom were adjusted using the Satthertwaite method. All the analyses were done with SAS (2001) [22].

## Results

**Figure 1:**
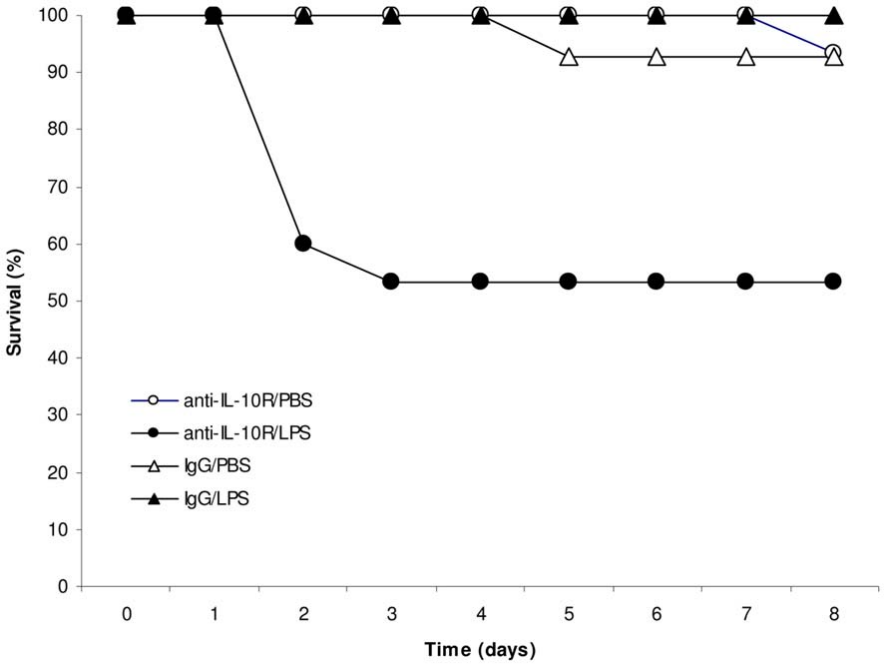
Survival rate of 15 month old mice during the course of the experiment for the two treatments. Empty dots: anti-IL-10R/PBS; full dots: anti-IL-10R/LPS; empty triangles: IgG/PBS; full triangles: IgG/LPS. None of the 3 month old mice died during the course of the experiment.

During the course of the experiment no young mice died (0/60), whereas 9 out of 58 old mice died (Fisher exact test, *P* = 0.0012). The analysis of the effect of the treatment on the survival was, therefore, restricted to aged mice. Old individuals treated with anti-IL-10R and with LPS suffered a substantially higher mortality rate compared to the other three groups (Log-Rank, χ^2^_3_ = 17.15, *P* = 0.0007, Figure 1), with mortality of anti-IL-10R/LPS mice occurring at days 2 and 3. The log-linear model further revealed that the interaction between the two treatments was statistically significant and that changes in body mass during day 0 and 2 did not predict survival prospects (table 1). Overall, these results show a strong effect of the suppression of the anti-inflammatory cytokine in old mice exposed to a mild inflammation, and suggest that mortality was not directly linked to changes in body mass.

**Table 1:** Log-linear model reporting the effect of the experimental treatments and body mass variation (percent variation in body mass between day 0 and day 2) on the survival rate of 15 months old mice.

*Source of variation*	*df*	*χ* ^2^	*P*
Percent variation in body mass	1	0.13	0.7162
Anti-IL-10R vs. IgG (Tr1)	1	4.07	0.0435
LPS vs. PBS (Tr2)	1	0.231	0.6332
Tr1 x Tr2	1	4.28	0.0386

**Figure 2:**
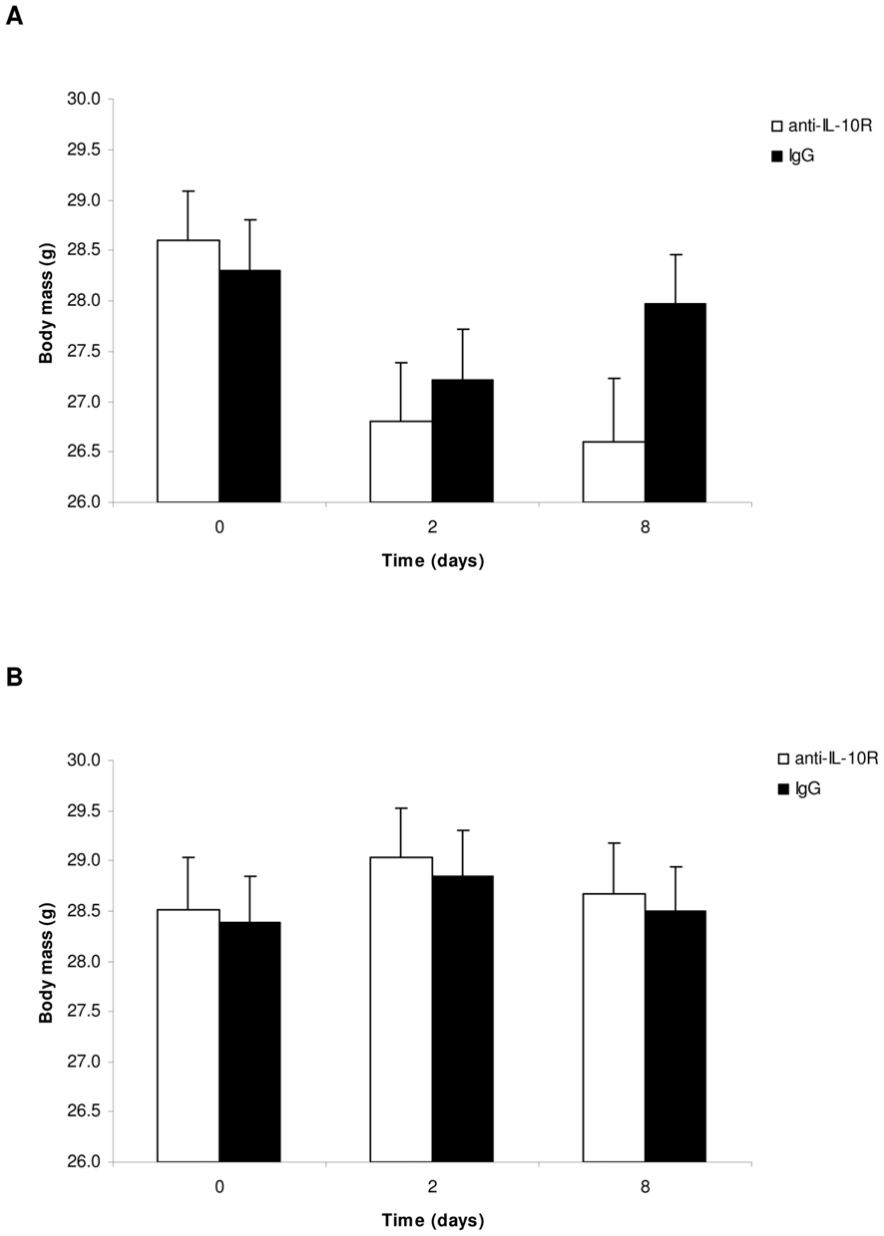
Temporal change in body mass of mice treated with anti-IL-10R antibodies or IgG. (A) LPS; (B) PBS. The bars represent the mean ± SE.

Changes in body mass during the course of the experiment depended on the interaction between the two treatments and the interaction between the anti-IL-10R treatment and age (table 2). As expected, LPS injection induced a quite substantial reduction in body mass, mass loss being even greater in the anti-IL-10R group (Fig. 2a). Body mass of PBS injected mice was stable during the course of the experiment (Fig. 2b).

**Table 2:** Generalized linear mixed model reporting the effect of the experimental treatments and age on the temporal variation in body mass (day 0, 2, 8).

*Source of variation*	*df*	*F*	*P*
Time	2,216	10.41	<0.0001
Anti-IL-10R vs. IgG (Tr1)	1,114	0.06	0.8048
LPS vs. PBS (Tr2)	1,114	12.21	0.0007
Age	1,114	213.84	<0.0001
Tr1 x Time	2,216	3.09	0.0476
Tr2 x Time	2,216	41.52	<0.0001
Age x Time	2,216	1.24	0.2919
Tr1 x Tr2	1,114	0.73	0.3939
Tr1 x Age	1,114	0.86	0.3552
Tr1 x Tr2 x Time	2,216	4.36	0.0140
Tr1 x Age x Time	2,216	3.81	0.0236

Mouse identity was fitted into the model as a random factor as to take into account the repeated nature of the data.

**Figure 3:**
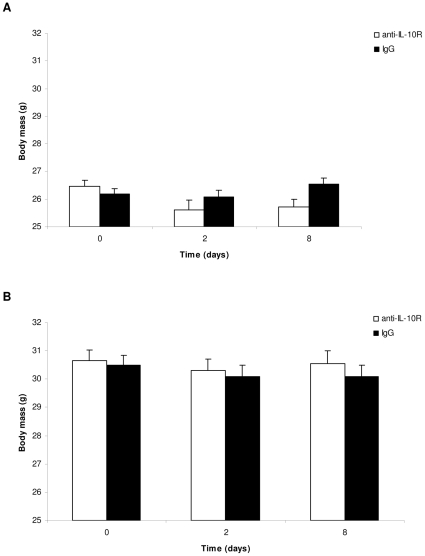
Changes in body mass of mice treated with anti-IL-10R antibodies or IgG. (A) young individuals; (B) old individuals. The bars represent the mean ± SE.

Anti-IL-10R treated young mice suffered from a reduction in body mass, whereas control (IgG injected) individuals had stable body mass (Fig. 3a). On the contrary, the anti-IL-10R treatment had no effect on body mass variation of old mice (Fig. 3b). This result is not the consequence of selective disappearance of old mice with the strongest body mass decrease because i) as shown above, mortality occurred independently from changes in body mass; ii) the same results were obtained when restricting the analysis of body mass variation on day 0 and 2 (i.e., when all mice were still alive).

**Figure 4:**
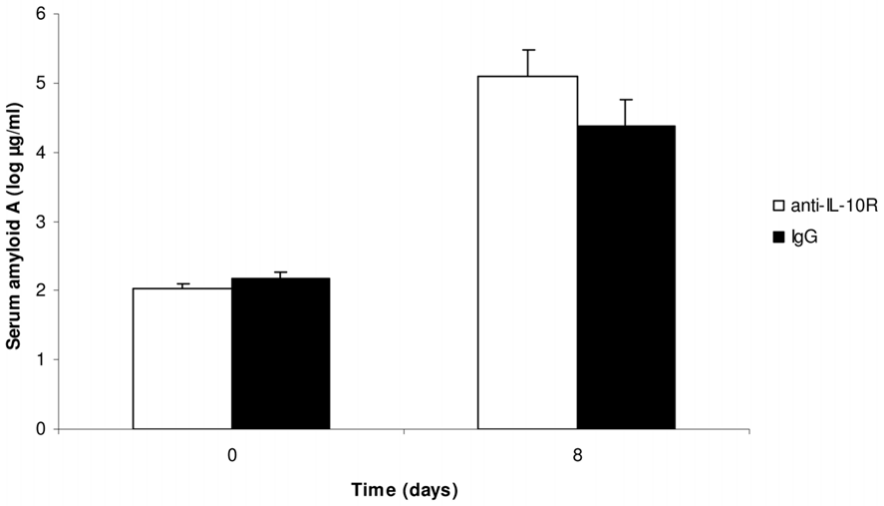
Effect of the anti-IL-10R treatment on the production of plasmatic serum amyloid A. The bars represent the mean ± SE.

**Figure 5:**
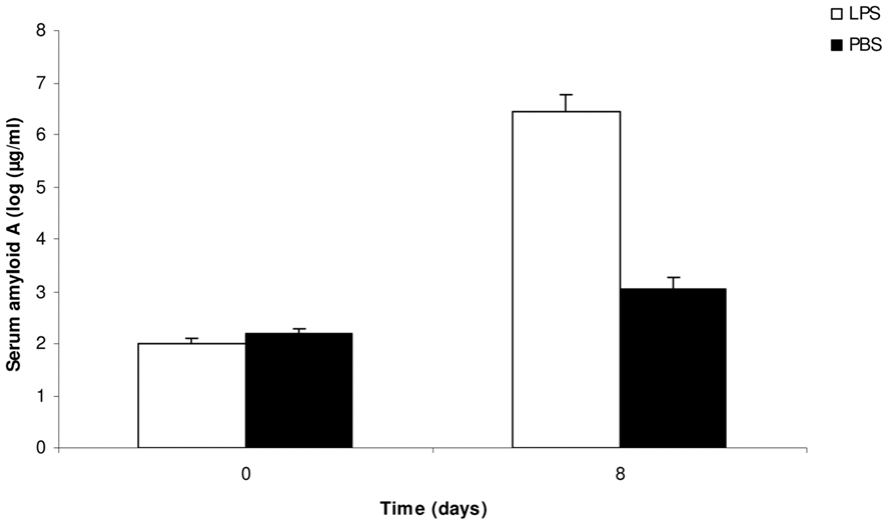
Effect of the LPS treatment on the production of plasmatic serum amyloid A. The bars represent the mean ± SE.

Both treatments had a strong effect on the production of the serum amyloid A (SAA), a protein of the acute phase response (table 3). The anti-IL-10R injection induced an increased concentration of plasmatic SAA compared to controls (Fig. 4), as did the injection of LPS compared to PBS (Fig. 5). Interestingly, however, age did not modulate the SAA production in response to the two treatments (table 3).

**Table 3:** Generalized linear mixed model reporting the effect of the experimental treatments and age on the temporal variation (day 0, and 8) in the plasma concentration of the serum amyloid A.

*Source of variation*	*df*	*F*	*P*
Time	1,151	203.88	<0.0001
Body mass	1,151	0.54	0.4628
Anti-IL-10R vs. IgG (Tr1)	1,151	3.086	0.0814
LPS vs. PBS (Tr2)	1,151	73.55	<0.0001
Age	1,151	0.36	0.5488
Tr1 x Time	1,151	5.88	0.0165
Tr2 x Time	1,151	93.24	<0.0001

Body mass at day 0 and 8 was also included as a covariate in the model. Mouse identity was fitted into the model as a random factor as to take into account the repeated nature of the data.

## Discussion

Here, we explored the fitness consequences of inhibiting an anti-inflammatory cytokine both under a mild inflammatory challenge and control conditions. Our findings show that suppressing the anti-inflammatory regulation leads to high mortality. Interestingly, however mortality only occurred in conjunction with the activation of the inflammatory response and in aged individuals. Although the treatment was repeated three times during the course of the experiment, mortality of LPS/anti-IL-10R treated mice only occurred following the first injection, suggesting that the treatment selected individuals with a particular pro- *vs*. anti-inflammatory profile [23]. Young individuals also suffered from the suppression of the immune regulation, since they experienced a reduction of body mass. However, this body mass loss was transitory and did not lead to any mortality.

While most ecological immunology studies implicitly assumed that evolution has shaped immune defences through resource-based trade-offs with other competing crucial traits [2], [3], our findings show that immunopathology can generate substantial costs, directly impairing survival prospects. Previous work has already put forward the potential role of autoimmune disorders in the evolution of immune defences [6]–[8], [24]. However, to our knowledge, this is the first study showing strong age-dependent survival cost of immune regulation suppression. This finding corroborates the view that immune regulation is a key process that should be considered when investigating the outcome of immune activation and its effects on host fitness.

Our results show that age is a very important factor affecting the cost of inhibiting immune regulation. Immunosenescence refers to the age-associated changes in immune functioning that are observed in humans and animal models [25], [26]. Immunosenescence does not necessarily describe a decline in immune performance but rather a series of changes in immune effectors that can finally impair immune protection. Longitudinal studies of elderly humans have shown that some immune markers can be reliably used as predictor of short term (2-year) mortality [27]. These markers define the Immune Risk Phenotype (IRP) which is characterized by a depletion of CD95- virgin T cells and the expansion of CD28- T cells, resulting in an overall reduced T cell repertoire in old ages [28]. The IRP is, however, also characterized by an increase of pro-inflammatory cytokines (such as IL-6 and TNF-α) and a low grade, chronic inflammation [29]. Our finding that old mice paid a severe cost when the regulation of the inflammatory response was experimentally impaired is in agreement with these previous results and further stresses that increased susceptibility to inflammation is a reliable marker of immunosenescence.

One of the central tenets of ecological immunology is that natural selection shapes the optimal investment into immune defences as to maximize fitness. Over investment into immune defences might be maladaptive if this diverts resources from other vital functions; similarly, a too small investment might make hosts too vulnerable to parasite exploitation. We think that another dimension has to be added to this simple trade-off. In addition to the total investment into immunity, selection is likely to strongly act on how well this function is regulated. Ignoring immune regulation might, therefore, provide a misleading picture on the real target of selection. For instance, an individual with a low total investment into immunity but with poor regulation might pay higher cost of immune activation than an individual with a huge allocation into immune defences and a very effective immune regulation. Clearly, ecological immunology needs to explicitly take into account immune regulation to better assess the selection forces acting on immunity.

Cost of reduced immune regulation, as shown in this study, suggests that natural selection has the potential to mold this trait. However, the strength of natural selection progressively declines with age, because the fraction of old individuals in a given population is much smaller than of young animals. We used for our study young adults (3 month old) and old (15 month old) mice. Fifteen month old mice can be considered old [30], but still not fully senescent individuals since animals that survived the experiment were still able to successfully reproduce (Belloni et al. unpublished). In addition to mortality cost paid by old individuals, young mice suffered from a transitory loss of body mass. Although all of them successfully recovered from the body mass reduction, it should be kept in mind that this occurred in a benign lab environment with no predators and food ad libitum. Therefore, overall, we believe that our results indicate that natural selection likely act on immune regulation.

In this study we focused on one anti-inflammatory cytokine (IL-10). IL-10 has been extensively studied during the last decade [31]. On one hand, IL-10 plays a crucial role in the resolution of inflammation and as such reduces the risk of immunopathology when organisms face an infectious disease. On the other hand, IL-10 can impede parasite clearance by down-regulating the inflammatory response. This antagonistic action of IL-10 has been demonstrated in several studies where experimental suppression of IL-10 results in better parasite clearance and increased immune-borne damage [31]. For instance, mice infected with the malaria parasite *Plasmodium chabaudii* and whose IL-10 was experimentally neutralized experienced shorter time to death compared to infected/IL-10^+^ controls, showing that immunopathology substantially contributes to the overall cost of infection in this system [32]. On the contrary, IL-10 deficient mice infected with the West Nile virus are better protected from the infection and enjoy a better survival than control, WNV infected mice [33]. These two examples illustrate how the costs and benefits of immune regulation are largely affected by the specific pathogens involved and how the immune system responds to them. However, to our knowledge, how age modulates the fitness costs and benefits of immune regulation has been largely overlooked.

To conclude, we report here experimental evidence showing that immune regulation is crucial for survival in aged individuals under a mild inflammatory challenge. To our view, this result opens a novel area of research on the fitness consequences of inter-individual variation in immune regulation.
